# Guidance for Implementing Video Consultations in Danish General Practice: Rapid Cycle Coproduction Study

**DOI:** 10.2196/27323

**Published:** 2021-11-24

**Authors:** Amanda Sandbæk, Line Due Christensen, Lotte Lykke Larsen, Nina Primholdt Christensen, Frida Greek Kofod, Ann Dorrit Guassora, Camilla Hoffmann Merrild, Elisabeth Assing Hvidt

**Affiliations:** 1 Research Unit for General Practice Aarhus Denmark; 2 Department of Public Health Aarhus University Aarhus Denmark; 3 Center for General Practice at Aalborg University Aalborg Denmark; 4 Research Unit of General Practice Department of Public Health University of Southern Denmark Odense Denmark; 5 Department of Hematology Odense University Hospital Odense Denmark; 6 The Research Unit and Section of General Practice Department of Public Health University of Copenhagen Copenhagen Denmark

**Keywords:** general practice, remote consultation, implementation science, resource guide, communication, video consultation, coproduction, rapid analysis, workshop, intervention development

## Abstract

**Background:**

The COVID-19 pandemic has changed various spheres of health care. General practitioners (GPs) have widely replaced face-to-face consultations with telephone or video consultations (VCs) to reduce the risk of COVID-19 transmission. Using VCs for health service delivery is an entirely new way of practicing for many GPs. However, this transition process has largely been conducted with no formal guidelines, which may have caused implementation barriers. This study presents a rapid cycle coproduction approach for developing a guide to assist VC implementation in general practice.

**Objective:**

The aim of this paper is to describe the developmental phases of the VC guide to assist general practices in implementing VCs and summarize the evaluation made by general practice users.

**Methods:**

The development of a guide for VC in general practice was structured as a stepped process based on the coproduction and prototyping processes. We used an iterative framework based on rapid qualitative analyses and interdisciplinary collaborations. Thus, the guide was developed in small, repeated cycles of development, implementation, evaluation, and adaptation, with a continuous exchange between research and practice. The data collection process was structured in 3 main phases. First, we conducted a literature review, recorded observations, and held informal and semistructured interviews. Second, we facilitated coproduction with stakeholders through 4 workshops with GPs, a group interview with patient representatives, and individual revisions by GPs. Third, nationwide testing was conducted in 5 general practice clinics and was followed by an evaluation of the guide through interviews with GPs.

**Results:**

A rapid cycle coproduction approach was used to explore the needs of general practice in connection with the implementation of VC and to develop useful, relevant, and easily understandable guiding materials. Our findings suggest that a guide for VCs should include advice and recommendations regarding the organization of VCs, the technical setup, the appropriate target groups, patients’ use of VCs, the performance of VCs, and the arrangements for booking a VC.

**Conclusions:**

The combination of coproduction, prototyping, small iterations, and rapid data analysis is a suitable approach when contextually rich, hands-on guide materials are urgently needed. Moreover, this method could provide an efficient way of developing relevant guide materials for general practice to aid the implementation of new technology beyond the pandemic period.

## Introduction

### Background

When COVID-19 was declared a pandemic by the World Health Organization in March 2020, face-to-face consultations were largely replaced by telephone, email, or video consultations (VCs) in general practice in Denmark, as in many other Western countries, to reduce the risk of COVID-19 transmission [1]. These changes were recommended by the Danish Regions and the Danish Organization of General Practitioners [2].

Although the sudden implementation of VC was triggered by necessity, remote communication between patients and health care providers has been recommended as a sustainable solution for some health service deliveries to ensure increased access to health care [3,4]. VC has been known to save time and reduce transportation costs for patients, to be suitable for patients unable to visit the clinic, and to be advantageous for patients who are uncomfortable with visiting a clinic (eg, patients with certain mental health problems) [3,5-7]. However, VC had only been used sparingly in general practice in Denmark before the COVID-19 pandemic [8,9].

To enable VC in general practice, the Danish Organization of General Practitioners developed access to VC through the mobile app *My Doctor* at the beginning of the COVID-19 pandemic. The *My Doctor* app is a multifaceted tool for patient–provider communication. Besides facilitating VCs, the app includes the possibility of renewing prescriptions, keeping track of appointments, and performing email consultations. The app is available free of charge for all general practitioners (GPs) and their patients in Denmark [10,11]. The mobile app provided increased opportunity for use, and the number of VCs during the lockdown (March 2020) rose abruptly to 23,500 per week from approximately 0 before the lockdown. This number decreased to approximately 6000 VCs per week after the lockdown [12]. The uptake of VCs in general practice is dependent on a range of factors, and context-adequate guidance on how to implement and conduct a VC is one of many [3]. Developing guide materials may support those who plan for continued use and those who wish to embark on using this new consultation form beyond the COVID-19 period [12,13].

### Objective

Research has demonstrated that a rapid cycle participatory design—involving stakeholders and end users in the development process—should be applied to ensure an efficient and agile approach. Using a participatory design provides a unique opportunity for developing a guide based on stakeholders’ and end users’ knowledge and experiences [2,13,14]. This design approach may result in a quickly developed guide that is conducted in a realistic setup and that takes the opportunities and constraints that already exist within this specific context into consideration [15-17]. Moreover, research focused on developing tools and guides in a realistic setup adds further knowledge of methodological considerations needed to guide the development of future health care solutions [18].

The aim of this study is to rapidly develop a tool to assist general practice (ie, GPs and practice staff) in implementing VCs in daily practice. Therefore, we have developed a guide on how to implement VC in general practice in Denmark through a rapid cycle participatory design. In this study, we present the development process, methodological considerations, and evaluations made by general practice users.

## Methods

### Setting

Danish health care is mainly funded by public taxes, with free-of-charge access to its services. General practice in Denmark is privately owned by GPs and organized into small units, either as single-handed practices (1 GP) or group practices (2-10 GPs), and almost every clinic has staff members. General practice is mostly financed through the public health care reimbursement scheme, and their services are regulated by collective agreements between the Danish Regions and the Organization of General Practitioners in Denmark [19,20]. At the beginning of the COVID-19 pandemic, a financial agreement was made for general practice to enable remuneration for the use of VCs [21]. General practice receives 165.27 DKK (US $25,67) per VC.

### Study Design

**Figure 1:**
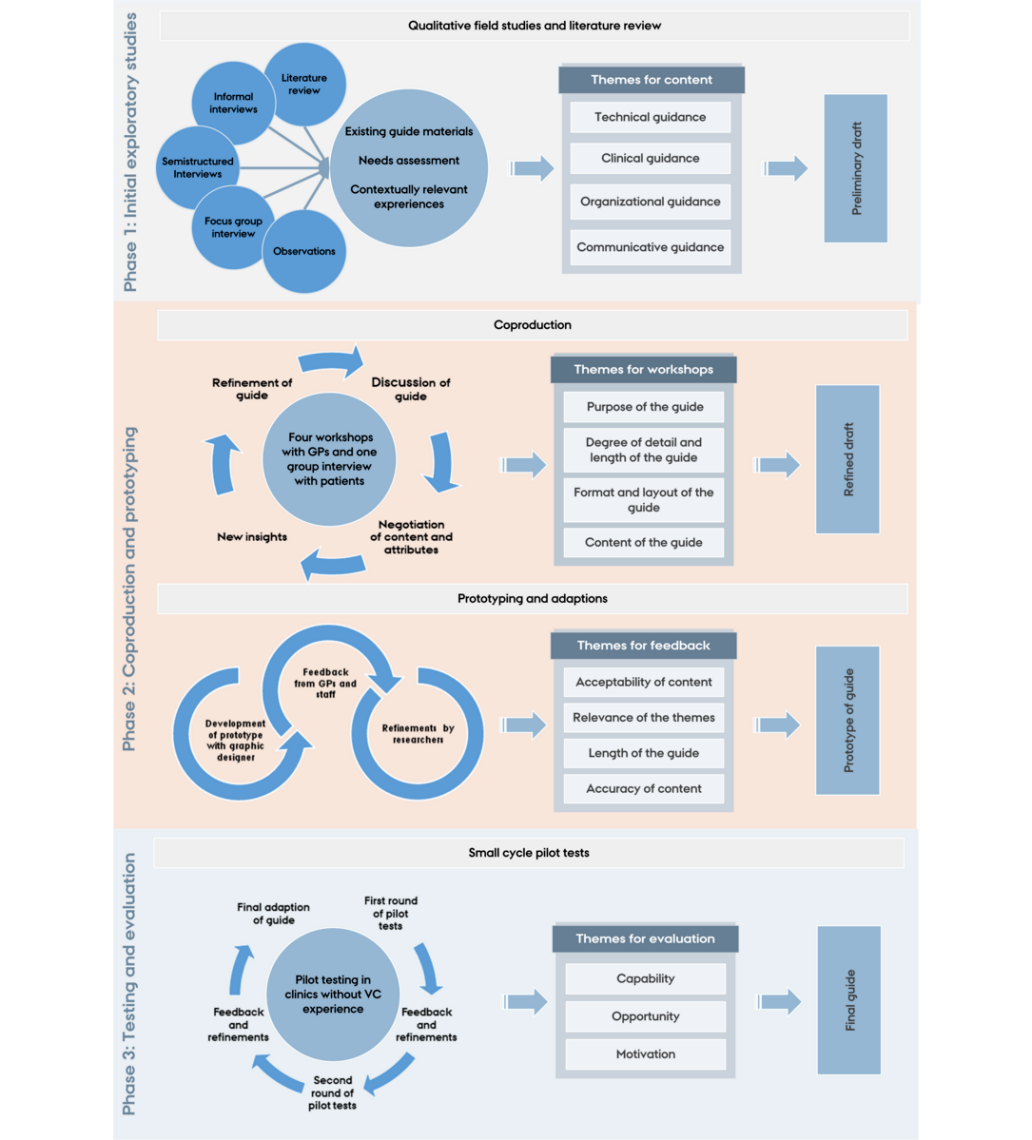
Overview of the framework and activities of the development of the guide. GP: general practitioner; VC: video consultation.

The development of the guide for general practice involved elements from coproduction approaches [22,23] and rapid cycle research [24]. This entails an approach in which researchers and end users collaborate throughout the project, using an iterative framework based on rapid qualitative analysis and interdisciplinary collaboration with a continuous exchange between research and practice. The study was undertaken in 3 phases comprising initial exploratory studies, coproduction, and evaluation, as illustrated in Figure 1. At different stages of the research process, insights from earlier phases were incorporated [25].

### Analysis Team

The interdisciplinary analysis team conducting the ongoing analysis (AS, LDC, LLL, FGK, and NPC) comprised researchers with different backgrounds, including public health (AS and LLL), health science (NPC), medicine (FGK), and pharmaceutical sciences (LDC).

### Phase 1: Initial Exploratory Studies

**Figure 2:**
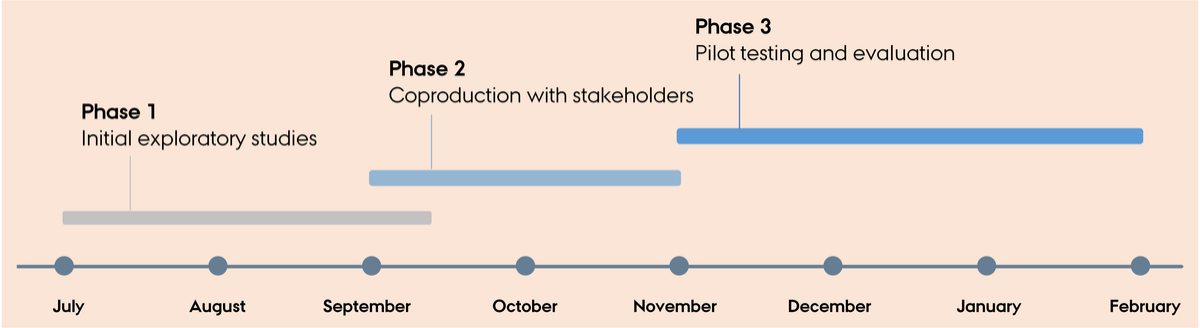
Timeline of the development process.

Phase 1 was conducted from July to September 2020 (Figure 2). We conducted a broad, explorative literature review, searching both academic and gray literature [26] on existing telemedicine guides, including telephone consultations and VCs. A total of 17 guides were identified but included only if found suitable for the Danish general practice setting. For instance, guides were excluded if they were concerned with telecommunication exclusively between health care providers, focused on legal- or insurance-related aspects of telecommunication or specific technical programs, were too lengthy, or merely repeated points from other identified guides.

To identify the need for guidance, ensure relevance and acceptability of the guide, and reduce problems with implementation, we collected primary data through observations and informal and semistructured interviews with 7 full-time GPs, 3 practice staff, and 2 patients (performed by AS and LDC). The participants were identified from the authors’ professional networks to foster a rapid first round of coproduction. Participants were not introduced to the research project before participation. Furthermore, a focus group interview was conducted with 7 data consultants, who were the technical or administrative staff charged with the role of providing technical and administrative support to general practice, for example, with VC implementation. The participants were purposefully sampled. This approach was used to select participants with broad experience in the field of VC. On the basis of the findings from the secondary and primary data, a first draft of the guide was developed.

### Phase 2: Coproduction With Stakeholders

#### Overview

The coproduction phase entailed 4 workshops with GPs and 1 group interview [27] with patient representatives. Workshops and group interviews were undertaken to develop the content of the draft guide developed in phase 1. The results from phase 2 were condensed into a prototype of the guide. The prototyping of the guide content was used to identify issues with acceptability and feasibility at an early point in development and allow them to be addressed before testing and evaluation [25].

#### Data Collection and Analysis

Phase 2 was conducted from September to November 2020 (Figure 2). The 4 workshops took place in 4 large cities in different regions in Denmark. The locations of the workshops in the large cities were chosen for convenience purposes but included GPs from across the country to ensure geographical variation. The participants were purposefully sampled based on their level of experience with VC. The number of participants ranged from 3 to 8 GPs in each workshop, which totaled 21 GPs (Table 1). All GPs had some or extensive experience with VCs. Workshops were used to discuss which key elements should be included in the guide to ease the implementation of VCs in general practice.

**Table 1:** Overview of participants (codesigners; N=54).

Type of participant	Phases	Number of participants	Gender (female), n (%)	Age interval, minimum-maximum
GPs^a^	1-3	32	16 (50)	36-71
Data consultants^b^	1	7	3 (43)	32-66
Practice staff	1-3	6	5 (83)	—^c^
Patients	1-2	4	1 (25)	39-58
National and regional stakeholders^d^	1	5	5 (100)	—^c^

^a^GP: general practitioner.

^b^Technical or administrative staff with a supporting role to help general practice with technical issues. The data consultants are placed in different regions in Denmark.

^c^Not possible.

^d^Leaders or employees working in regional or national health institutions, that is, prehospital emergency medical services, regional quality development units, and general practice support units.

The workshops comprised individual and group activities commonly used in user experience design [28], such as reflection exercises, discussions, negotiations, and polls. All activities included graphic facilitation with active engagement, visualizations, or physical manifestations to provide a shared nexus for communication. The workshops were facilitated by AS and LDC. The aim was to not only encourage participants to draw on their own VC experiences, creativity, and expertise but also to reflect on their own needs in the early implementation stage, as the end users of the guide would be inexperienced users. The facilitators led the GPs through 4 separate processes involving reflections on the barriers and facilitators related to the use of VC in general practice, discussions on how to organize VCs in the existing general practice setting, and discussions on when to use VC instead of face-to-face consultation. Finally, the layout of the guide was discussed. Sufficient information power was obtained [29].

We recruited 2 patient representatives with significant knowledge on patient safety and patient–physician communication through the Danish Society for Patient Safety, and they participated in a group interview. They were asked to focus on the VC guide from their patient perspectives, and the themes and results of the GP workshops were presented to them. This allowed the patient representatives to provide inputs on how GPs and practice staff should implement VCs; for example, advice and knowledge regarding potential pitfalls in terms of ensuring patient safety and satisfaction with VC.

A total of 2 researchers (AS and LDC) from the analysis team and a research assistant reviewed the video recordings from the workshops, the participants’ notes, and the researchers’ notes from the workshops and group interviews. Subsequently, in collaboration, the researchers made iterations based on the open and axial coding of the participants’ comments on the content in the guide, and new aspects were incorporated [30,31]. Data from one workshop were used to develop a preliminary codebook of predefined codes concerning the elements for the guide materials. On the basis of this codebook, each member of the analysis team analyzed parts of the workshop data and group interview data. Afterward, the analysis team discussed the identified themes and positions across the initial analyses to obtain consensus on the content of the guide, which was to be included in the new prototype of the guide [30,31]. The analysis team used NVivo (version 13; QSR International) software to manage and code all the data.

We invited former workshop participants, GPs, and practice staff from phase 1 (Table 1) to review the newly developed prototype of the guide in the form of a webpage. The review process was based on a rapid cycle improvement concept and included several small circles of reviewing of both the layout and components of the guide [32]. Participants were asked to assess 5 features: the acceptability of the guide materials in daily clinical practice, the relevance of the selected themes, the length of each section (considering the relevance of the particular theme), the accuracy of the information, and the format (whether the materials were presented in a convenient format with simple, clear, and easy-to-understand messages). A total of 2 researchers (AS and LDC) from the analysis team evaluated the statements and incorporated the adjustments that were widely agreed upon or, in other ways, deemed generic.

### Phase 3: Pilot Testing and Evaluation

Phase 3 was conducted from November 2020 to February 2021 (Figure 2). After iterations of development and refinement, the prototype was tested by GPs and practice staff who had not been involved in the development phases and had little or no experience with VC. These participants were selected to represent the end users of the guide. A total of 7 semistructured interviews were undertaken by NPC. In total, 5 clinics from 4 different Danish regions pilot-tested the guide. The participants were purposefully sampled and had access to the materials for approximately 2 months. During this period, they were asked to use the materials in daily practice and follow the proposed steps. Subsequently, 4 GPs and 3 practice staff members from these clinics participated in the evaluation interviews (Table 1). The interviews were conducted via telephone, audio recorded, and based on a semistructured interview guide that was inspired by the theory of capability-opportunity-motivation and behavior (COM-B) model, which proposes that people need capability (C), opportunity (O), and motivation (M) to perform a behavior (B) [33]. The COM-B model guided the structure of the interview guide into questions relating to capability (how and to what extent does the guide help GPs and practice staff obtain the required knowledge and skills in relation to VC implementation), opportunity (how and to what extent does the guide help to obtain and facilitate the use of time resources, equipment, and support properly), and motivation (how and to what extent does the guide help motivate VC implementation reflected through prioritization and emotional drive). Furthermore, the COM-B model was used as a theoretical framework for analyzing the data for the final revision of the guide materials [33]. Meaningful units related to the components of the COM-B model were identified and thematically coded. The analysis was also based on the rapid analysis approach [34], and iterative refinements were made during pilot testing [16]. Thematic coding was performed by AS, LDC, and NPC, and emerging themes were discussed with the other authors. This iterative process led to an agreement on the final version of the guide, which was adjusted upon completion of the pilot testing and evaluation phases.

### Ethics

No approval from an ethical committee was needed for research involving observations, interviews, and workshops according to the national research guidelines in Denmark [35]. This study was registered in the Record of Processing Activities at the Research Unit for General Practice, Aarhus, in accordance with the provisions of the General Data Protection Regulation [36]. All participants from the workshops, focus group interviews, and face-to-face interviews gave written informed consent. Participants gave oral informed consent before participating in observations, informal interviews, or web-based interviews. The study complies with the Helsinki Declaration [37], and data storage and access comply with the General Data Protection Regulation.

## Results

The results from the development of the guide have been described in the following subsections and illustrated in Figure 1.

### Phase 1: Initial Exploratory Studies

We identified 17 documents containing guiding materials for VC from Denmark (11/17, 65%), the United States (1/17, 6%), Australia (1/17, 6%), and England (4/17, 24%). We included 3 guides [38-40] that were used as secondary sources for developing the content for the new guide in this study. This condensation was based on the considerations regarding relevance, acceptability, and potential to construct a meaningful guide. The primary data showed that GPs with minimal or no experience were hesitant to use VC in practice, needed knowledge and experience with VC, experienced clinical, organizational, and technical barriers, were confused regarding where to begin, and requested guidance for implementing VCs. The data consultants expressed similar needs and barriers based on their knowledge of aiding GPs to start with VCs. In addition, data from VC-experienced GPs and staff helped identify the potential steps for the implementation of VCs related to workflow, organization, communication, and division of tasks. On the basis of these qualitative explorative studies and the existing literature, we identified 4 fundamental needs for guidance to successfully implement and promote VC in general practice: technical guidance, organizational guidance, communicative guidance, and clinical guidance. To meet these needs, we constructed 3 components inspired by the 3 international guides identified in the literature: a gross list of potential content themes, an initial prototype of the guide, and a *toolbox* comprising communication phrases and a list of medical conditions deemed relevant for VCs. All 3 components were to be considered and discussed in the following workshops (phase 2).

### Phase 2: Coproduction and Prototyping

On the basis of a qualitative team coding analysis across the 4 GP workshops and the group interview with patient representatives, 7 main themes were constructed: *format of the guide*, *implementation of VC*, *organization*, *communication*, *competencies*, *technical issues*, and *medical conditions*. These main themes represent important aspects to consider when developing useful, relevant, and easy guiding materials.

#### Format of the Guide

The layout of the guide was a highly discussed topic during the workshops and was adjusted accordingly. GPs preferred short and concise guides without heavy text. They preferred the guide to not exceed 1 page. Some GPs suggested that the guide be presented as bullet points:

It is like when you buy a device at home, then it comes with a gigantic instruction manual that nobody reads. And then there are the five bullets in the “How to” instructions that everybody reads, and that is how it should be since both opportunities are required. But for those who are just about to start, there should be no more than 10 bullets.

GP 11

Some GPs mentioned that they preferred colorful guides, and others emphasized that guides in the form of a postcard (hard copy 1-pager) were appealing. In addition, although a few GPs cherished the option of a hard copy edition, GPs generally agreed that the guide should be available on the web. The GPs had many suggestions for content, which contradicted the idea of a short format and argued for dividing the guide into a short and a long version. Some suggested a web-based guide with more detailed information and materials for the implementation of VC, whereas the hard copy should be in the form of a checklist.

#### Implementation

According to GPs, a VC guide should address both the one-off challenges expected to occur during implementation, such as the technical aspects of setting up VCs, and the recurring challenges related to the development of routines, workflow, and division of labor. Moreover, GPs discussed how these barriers could be met during implementation. Some GPs experienced barriers related to the identification of relevant guidance, whereas others highlighted the significance of motivating practice staff for its use:

It is cooler when they [practice staff] take part in it, and are dedicated and can see the point of it.

GP 8

#### Organization

The organizational aspects included structuring, planning, and coordination of activities related to VC. A substantial difference in the organizational structure was identified between the general practices, challenging the development of advice regarding a general, recommended organization of VC. GPs generally supported the idea of an introductory staff meeting before VC implementation, where the collaboration and workflow related to the implementation of VC could be discussed. Similarly, GPs endorsed the idea of a scheduled evaluation meeting in clinics, as a continuous exchange of experiences was not self-evident. Themes for both introductory and evaluation meetings were proposed and discussed:

I also believe it is important when implementing new things that you follow up on it. That is always the case, not only in video consultations, but I believe that it’s very, very important, especially when we find something difficult, to evaluate and solve the problems that may otherwise make you decide not to use it.

GP 9

Moreover, GPs raised concerns regarding the organization of new workflows for referring patients to VCs, as the practice staff was not confident regarding the new visitation possibilities. Thus, an example of how the visitation could be organized was incorporated into the guide.

#### Communication

The communicative theme comprised both 1-way (typically written) and 2-way (typically oral) communication among GPs, staff, and patients, for example, on the clinic’s website and during consultation. Several GPs expressed that patient communication before and during the VC was challenging and requested guidance on this topic to ensure a safe environment for both the patient and the GP. According to both patients and GPs, patients also needed guidance before the consultation on, for example, booking, technical issues, preparation, and what to expect. In particular, the role of practice staff in communication with patients was mentioned as an important topic for the successful management of VCs:

When COVID-19 was at its worst, we had a lot of them [video consultations] every day. This number has gone down now, and I think that it is because the receptionist doesn’t really remember it, and the patients don’t really know that it’s an option.

GP 10

#### Competencies

It became apparent that practice staff has a key role in relation to the organizational aspects and the booking of VCs. Thus, the continuous education of practice staff regarding their role and potential tasks in VC seemed advantageous. Although the GPs generally felt capable of performing a VC, several GPs benefited greatly from sharing experiences regarding the use of VC with other participants during the workshop. However, a *trial and error* approach was acknowledged as a common approach, and 1 GP mentioned that GPs often tried to solve the problems themselves, although guides were available. Moreover, a general opinion on the subject of *when and for whom VC is relevant* was that the best way to learn was through practical experience:

I need to throw myself into things and get some experience with a lot of things, where the mistakes may happen before I even realize what it concerns. If I do that [get knowledge about video consultation] in advance and don’t get to use it until a month later, then I will have forgotten what I prepared anyway.

GP 19

Thus, GPs generally thought that enhancing the competencies for VC among GPs and the practice staff required more than a guide, as their competencies were determined by the amount of practice and time spent. However, nearly all the participating GPs recommended each practice to designate a VC *super user* among the employees in their clinic to aid colleagues in solving the problems encountered during the implementation and use of VCs.

#### Technical Issues

Most GPs expressed that they had experienced frustrating technical challenges when using the *My Doctor* app, such as problems regarding the booking of a VC, the web-based waiting room, the interaction between the medical software systems and the *My Doctor* solution, and the possibility for relatives to participate in a VC. Some said that technical challenges often constituted the main barrier to implementation, as most GPs did not consider technical skills a core task and thus easily lost their patience and turned toward familiar solutions, which would typically be face-to-face consultations:

If a general practice experiences problems with video consultation, they will quickly give up and never really get started.

GP 1

Therefore, GPs suggested that a guide for implementation should address how to overcome the most common technical challenges and offer step-by-step advice on how to get through the technical challenges. Moreover, the guide should clearly state where to seek more information or help in the case of technical difficulties.

#### Medical Conditions

Medical conditions deemed relevant for VC and listed as part of the initial version of the guide were debated in the workshops. The relevance of VCs in general practice is dependent on a range of factors, which was also pointed out by the GPs in this study. Some GPs requested a list of conditions that were relevant for visitation to VC (for inspiration), whereas others expressed that a list of conditions suitable for VC would be too narrow and dichotomous. The GPs were concerned about compromising their professionalism by adhering to a list when planning for a VC, as it would never be comprehensive enough to encompass all potential situations. Therefore, they suggested that visitation should always be an individual assessment. However, a significant barrier to the initiation of VCs was the identification of medical conditions suitable for a VC. A compromise agreed upon by the GPs was to recommend practices to start the VC implementation process by inviting well-known patients with relatively simple inquiries (eg, a status consultation with a familiar patient with well-regulated diabetes) to enable GPs to become gradually more comfortable with the use of VC without compromising patient safety. A GP explained it as follows:

We took the really easy patients and the really easy diagnoses, and then slowly added more and more.

GP 8

#### Patients’ Perspectives

A total of 4 main points were extracted from the group interviews with the 2 patient representatives. First, they argued that the GP should not expect patients to book a VC as patients are not able to assess whether a VC would be suitable in their case and would, therefore, typically resort to the familiar form of consultation. Second, adequate patient communication is important to minimize misunderstandings and ensure patient safety. Third, the GP should inform the patient that a physical consultation would always be an option if necessary. Fourth, the GP should explain in detail to the patient what will happen during a VC. These results were incorporated into the guide.

#### Prototyping

On the basis of the workshops and group interviews, a prototype was developed in an iterative process with stakeholders. The process resulted in both linguistic and format corrections, which were decided by the analysis team. Elements that conflicted with daily clinical practice and technical details related to the setup for the *My Doctor* app were modified. The guide included sections on *how to get started* and *daily activities related to VC*. These were divided into 7 main steps: determining the organization of VCs, testing the technical setup of VCs, deciding how to use VCs, preparing patients to use VCs, performing a VC, learning from other GPs’ experiences with VCs, and setting up a standard way of booking a VC. An additional section targeting the practice team was included in the guide; this section focused on how to evaluate the suitability of a patient’s health problems for a VC and the administration of patient communications, appointments, and bookings. Several linguistic edits were made to reduce technical ambiguities. Finally, the guide was split into 2 documents: a full-length guide (webpage and downloadable file) and a checklist (downloadable file).

### Phase 3: Testing and Evaluation

Interviews with GPs and practice staff conducted upon pilot testing showed that the clinics had used the materials differently. Some had followed the steps thoroughly and succeeded in performing several VCs with their patients, whereas others had implemented selected parts and conducted a few VCs. The following subsections will present how the GPs and staff experienced the guide’s ability to equip them with the necessary capability, disclose pathways to acquire the opportunity, and thereby foster the motivation that drives the implementation process.

#### Capability—Strengthening Skills and Knowledge

After being introduced to the guide materials, both GPs and practice staff generally agreed that the guide had improved their knowledge regarding how to implement VC and thereby their capability of performing a VC:

It [the guide] was like following an easy instruction manual. Well, it was clearly written, it was divided into nice sections, which also made it easy if you were in doubt about something because then you could just go to that particular section.

GP 22

However, GPs did not always use the materials as intended. It was common for the GPs to only read the checklist and instead rely on the practice staff to familiarize themselves with the combined materials (the webpage, guide, and checklist) because of time constraints. Several GPs pointed out that, by relying entirely on the checklist, they did not acquire the knowledge required to avoid frustrating challenges during the implementation process. During the interview session, some GPs recognized that they might have acquired the necessary knowledge base to avoid these frustrations if they had read the materials more thoroughly before performing the VCs. In cases where GPs found the guide materials insufficient, it appeared that the GPs were unaware of the relationship between the webpage, the guide, and the checklist. The limited knowledge of the relationship between the webpage, the guide, and the checklist could be a barrier to using the materials. In these situations, the overlapping materials (checklist and webpage) were experienced as redundant instead of helpful. However, both GPs who used the materials as intended and those who did not indicated that the guide had improved their overall capability of implementing and performing VCs in daily practice.

Accordingly, the practice staff who had used the entire materials expressed that the materials were sufficient and had conveyed the appropriate amount of knowledge for implementing VC. Furthermore, the guide was perceived to strengthen their skills in performing a VC, encouraging them to experience technical solutions from the patients’ point of view. A practice staff explained the following:

And then it is a really good idea this thing about trying it yourself so that you can see it from the patients’ point of view. This is extremely important.

Practice staff 1

Although the guide helped most of the practice staff and GPs gain knowledge regarding how to invite patients for VC, it was still found difficult to obtain the skills in some practices, because patients often declined to participate in a VC:

Unfortunately, there are not many patients who want to have a VC.

Practice staff 1

#### Opportunity—Facilitating Organization and Workflow

GPs and practice staff experienced that the guide could serve as help when organizing a VC and that it served as a tool to achieve the necessary structure in the clinic to be able to offer VCs, which contributed to increased implementation chances. The materials appealed to the readers at different experiential levels. For some clinics, it was the technical setup (eg, internet speed and equipment) that was most vital for facilitating sufficient opportunities to implement VC, whereas others perceived the guidance to facilitate the organizational framework (eg, how to set up patient bookings in the booking system) as the most important part:

Well, the technical part of making it work and testing it was easy enough. It was first when we came to “how do we introduce this [VC]” that it became difficult. At that point it [the guide] turned out useful.

GP 23

However, by nature, guides cannot facilitate the opportunity to ease the time pressure experienced in general practice. This challenge was addressed by many GPs, who reported a lack of time to become familiar with VC:

The guide is easy, but [creating] the routine is difficult. You have a busy schedule.

GP 23

In addition, GPs were not economically compensated for the time they used to read the guide materials, which, according to some of the GPs, made it difficult to justify that reading the guide materials should be prioritized over other important tasks in daily practice. Consequently, GPs preferred starting quickly and spending little time reading before starting:

The guide was too long, and as a doctor, you just want to get started, right? And [you] don’t have the time to read through the whole guide.

GP 24

However, some clinics dealt with time constraints by letting one of the practice staff explore the materials more thoroughly. The practice staff member explained that she had used the guide to improve their clinic’s home page, which increased the opportunity for VCs by increasing their accessibility to patients:

I needed some good advice on how to present the VCs at our homepage, and the guide was a big help. The GP used the checklist, and I used the long guide with the examples of phrases.

Practice staff 1

In contrast, 1 GP mentioned that having read the guide thoroughly could have saved her time, as she would have avoided spending time on the consultations unfit for VC. She elaborated the following:

It is great that somebody has spent a lot of effort thinking about “Who is it [that is suited for video consultation]” and “think about whom you want to book,” right?...I am a little like “no, let’s just get started,” and then some receptionists will deal with it [booking], and when they, for the fourth time, have booked a video consultation for somebody with a shoulder injury, then it’s a little annoying because it’s somewhat difficult to perform a physical examination of the shoulder through video.

GP 25

Facilitating the opportunity for VC implementation was challenged by the fact that most GPs struggled with organizational issues (eg, delegating the task of opening the web-based waiting room in the *My Doctor* app). Although many GPs and practice staff explained that the guide had supported the establishment of VCs, an exact *recipe* for using the web-based waiting room was not offered in the guide. Instead, it was put forward as an issue that the general practices should agree upon internally.

#### Motivation—Increasing the Motivation

Overall, the guide materials were perceived as acceptable, feasible, and relevant for the implementation of VC in general practice among both GPs and practice staff. The guide was described as useful, intuitive, and comprehensible. A common view was that it was easy to use and adopt in daily practice, which was described as a motivation to start:

Thank God, it [the guide] was easy. The guide made it much easier to get started with the VCs.

GP 22

Moreover, the guide increased the motivation to implement VCs because of its simplicity (layout and content). In particular, the practice staff found it motivational to use the materials on the webpage, as it was found to be directly transferable and applicable in daily practice. One GP mentioned that the division of the guide into clear sections made it easily approachable. However, another GP argued that ideally, the guide materials should have been divided into sections that are aimed separately at GPs and practice staff.

Although the guide materials encouraged the introduction of team meetings in the implementation process, GPs described how they first and foremost felt that they had the responsibility. Some GPs felt alone during the process of implementing VCs. Combined with time limitations within the organization, this meant that the GPs easily lost their motivation, which made it difficult to change their daily routines:

I wish it could be possible to share more knowledge with colleagues about VCs—I feel alone with the responsibility to get the VCs on track.

GP 25

Both GPs and staff expressed that being interviewed about the use of the guide and the checklist made them more motivated to start using VCs and use the guide more systematically for a good start. Taking the time to reflect on the guide materials acted as a motivational factor for the respondents. One GP explained that she would share her guide experiences with a colleague after the interview:

I will bring the checklist and the guide and all sorts of things to my colleague tomorrow so that she can try to get started too.

GP 23

GPs and the practice staff agreed that they would recommend the guide materials to other colleagues, and they thought that the guide had addressed the most important issues regarding the implementation of VC. Thus, in addressing capability and opportunity, the guide increased the motivation for implementing VC.

#### Final Adaptions

On the basis of the feedback in phase 3, minor adjustments were made to wording and format. The contents of the guide were then finalized. The final 7 main themes identified were as follows: determining the organization of VC, testing the technical setup of VC, deciding how to use VC, preparing patients to use VC, performing a VC, arranging bookings for VCs, and considering other useful advice [41].

Some GPs found that the guide comprised sections irrelevant to their specific situation, which ultimately made it too lengthy. This experience of relevance varied between GPs and across sections, making it difficult to shorten the guide. Therefore, we inserted a table of contents and customized the layout with clear headings to enable future users to easily decide upon the sections they found pertinent.

The pilot test indicated that GPs and practice staff were capable of adopting the VC guide to implement VC in general practice. In addition, a short introductory video was added to the webpage to underpin the relationship between the guide materials and support the implementation process.

## Discussion

### Principal Findings

A rapid cycle coproduction approach was used to explore the needs of general practice concerning the implementation of VCs. This approach was taken to develop useful, relevant, and easy guiding materials. The 3-phased framework used in this study provides a pragmatic example of coproducing and prototyping tools that fit into the everyday clinical workflow and meet the needs of general practice. The use of a small cycle iterative approach enabled us to address implementation issues at the design stage and modify the tool to overcome common barriers [25]. Our framework offers insight into how collaboration and coproduction with stakeholders can be incorporated into these different stages of intervention development. In codesign approaches, end users are involved throughout the development processes and work together with the research team during all phases [17,42]. In this study, GPs, practice staff, patients, and other stakeholders contributed to the development by generating content specific to the guide.

During data analysis and guide refinement, a rapid assessment procedure was used. This approach entails an in-depth understanding of the important elements for the guide without transcribing verbatim. This is a useful approach for creating comprehensive information in short timelines [43]. Our approach was similar to other rapid methods, such as using a priori structured codebook [44], coding audio only [45], and allocating researchers to code for specific themes [46]. Such rapid analysis approaches have been shown to produce valid findings, compared with traditional in-depth, line-by-line transcript analysis [34]. Thus, when conducting research with time constraints, this approach could be considered a supplement to the qualitative researcher toolkit [44].

The COM-B behavioral-inspired analysis [33] gave insights into how the guide was adapted and used in a clinical setting. GPs and practice staff felt equipped to implement VC after reading the guide. However, to implement VC in daily practice, the GPs needed to spend some time organizing VC in general practice. However, the guide motivated GPs and practice staff to implement VC. However, this implementation will not succeed solely by distributing the guide. Following the diffusion of innovation theory [47] and as widely recognized in the empirical literature, some clinicians adopt technological innovations readily, whereas others need motivation and support [13]. The guide in itself may not facilitate GPs gaining experience and confidence in VCs. Instead, GPs who are, by nature, not early adopters would benefit from a multifaceted intervention to aid the implementation of VC (eg, through peer training and the possibility of debating with colleagues having more extensive VC experience). Jensen et al [48] concluded that GPs need different approaches to implementation support and that the combination of support types depends on their needs and willingness to invest resources in future interventions. Moreover, a study from Australia [49] concluded that establishing VC as routine practice would need to be endorsed by patients, GPs, and founding organizations. Thus, proper complementary supportive interventions would be required to obtain the synergic effects of a multifaceted approach and fulfill the potential of the guide materials.

Resistance to change is common, and concerns relating to the introduction of alternative or supplementary methods of consultation can be expressed as concerns regarding patient safety. GPs and practice staff have responded to alternative forms of consultation with a mix of enthusiasm for innovation and resistance to change [7]. However, a guide developed with stakeholder engagement through coproducing methods could infuse less resistance, as peer professionals are listed as senders of the messages. Moreover, the guide addresses difficulties and concerns that have been empirically defined. However, the implementation is likely to be a difficult and resource-intensive task that would require both national and local strategic leadership [50].

The guiding materials were developed during the COVID-19 pandemic. These contextual factors may have influenced the development process, as many factors changed rapidly during the process [17]. This could affect the topicality of the guide during the postpandemic period. However, by having paid attention to the common usability of the guide across time (eg, by informing stakeholders to consider the VC guide from a general and decontextualized perspective), we consider the guide to be relevant beyond the COVID-19 context. Nonetheless, the guide is both applicable and particularly relevant in a COVID-19 context, as VCs may reduce the risk of COVID-19 contagion because of the reduced physical contact.

The use of VC during the COVID-19 pandemic may yield useful knowledge regarding which patients and diseases would be relevant for VC and whether the use of VCs may be extended to a wide variety of patients and clinical situations. Moreover, there is a long-term perspective on the use of VC and thus a strong need for a guide on its implementation. The population of older adults is increasing, and the aging population has an increasing number of health problems. This may further increase the pressure on primary health care, which is often the first point of contact. In Denmark and similar countries, access to GPs is a rising concern, especially as their unavailability may push some people to seek help elsewhere or not at all [51,52]. If quicker and easier access to care means that more people in need of care would contact general practice, VC could be adopted as a patient entitlement under universal health coverage [53]. However, offering VCs may also increase the workload in general practice unless the video-based encounter is of shorter duration than (or of similar length to) an ordinary face-to-face consultation and requires no subsequent consultation [53]. The provision of VC is likely to depend on the remuneration for this type of service, and the remuneration of GPs for services provided is regulated by collective agreements between the Danish Regions and the Danish Organization of General Practitioners in Denmark [19].

### Strengths and Limitations

A strength of this study is that all steps in the development process were described in detail. Using a coproduction approach with the triangulation of multiple data sources was an additional strength. Coproduction is known to improve the adaptation of the guide to a given context [25]. Using a coproduction approach allowed us to involve few but specifically selected participants for each phase. Concurrently, the rapid cycle approach ensured an agile process with adequate and suitable inputs.

Analysis of the qualitative data with a team of coders proved highly successful in terms of reaching as many different perspectives on the available data as possible. The inclusion of additional coders and their interpretations served to enhance the credibility of the framework that emerged.

In 2 out of 4 workshops, it was only possible to recruit 3 participating GPs in each workshop compared with the 8 and 7 participating GPs in the 2 other workshops. Furthermore, although the 2 workshops contained only 3 GPs, we assessed that they contributed with just as many diverse perspectives and nuances regarding the content of the guide as those provided in the workshops with more participants. To some degree, homogeneity is recommended for participatory design [14]. The heterogeneity in our workshops showed that GPs had different needs and preferences. Generally, the coproduction processes contributed to identifying many perspectives on and experiences with VC to improve the guide’s content.

General practice in Denmark is organized into different types of clinics. VC will be implemented and used differently in the clinics, and it can be difficult to adapt VC to all the clinics. However, in all phases of this study, the recruited GPs were initially selected to represent a variety of GPs with and without experience with VC [17]. All GPs and other stakeholders were purposely sampled across the nation. Another strength of the developed materials is that the implementation guide and the website are freely available in a format that is directly applicable to general practice.

A limitation was that we included only 2 patients in the analysis. Consequently, we must conclude that the patient perspective is only sparsely presented in the guide. However, the included patients were formally appointed patient representatives who were experienced in speaking on behalf of a broad patient population. Moreover, the documentation was challenged in the rapid analysis, which limited the controllability of the analysis, as the usual coding process was reduced to an oral discussion [54]. We addressed this by recording and revisiting the group discussion to enable tracking of the iterative analysis. The rapidity of our analysis implied that we did not progress into higher levels of abstraction and interpretation but rather focused on the potential improvements to the guide.

### Conclusions

Coproduction, involving prototyping, small iterations, and rapid data analysis, is a suitable approach when contextually rich, *hands-on* guide materials are urgently needed. The new guide for VCs was developed through rapid cycles, team-based work, and acknowledged research methodology. It comprised the following themes: organizing a VC in general practice, testing the technical setup of VC, deciding how to use VC, preparing patients to use VCs, performing a VC, arranging bookings for a VC, and proposing useful advice.

## Abbreviations

COM-B: capability-opportunity-motivation and behavior
GP: general practitioner
VC: video consultation
